# Transcriptomic response to differentiation induction

**DOI:** 10.1186/1471-2105-7-81

**Published:** 2006-02-17

**Authors:** GW Patton, R Stephens, IA Sidorov, X Xiao, RA Lempicki, DS Dimitrov, RH Shoemaker, G Tudor

**Affiliations:** 1 Screening Technologies Branch, Developmental Therapeutics Program, NCI-Frederick, Frederick, MD 21702, USA; 2 Advanced Biomedical Computing Center, NCI-Frederick, Frederick, MD 21702, USA; 3 Center for Cancer Research Nanobiology Program (CCRNP), Center for Cancer Research, NCI-Frederick, Frederick, MD 21702, USA; 4 SAIC-Frederick, Frederick, MD 21702, USA

## Abstract

**Background:**

Microarrays used for gene expression studies yield large amounts of data. The processing of such data typically leads to lists of differentially-regulated genes. A common terminal data analysis step is to map pathways of potentially interrelated genes.

**Methods:**

We applied a transcriptomics analysis tool to elucidate the underlying pathways of leukocyte maturation at the genomic level in an established cellular model of leukemia by examining time-course data in two subclones of U-937 cells. Leukemias such as Acute Promyelocytic Leukemia (APL) are characterized by a block in the hematopoietic stem cell maturation program at a point when expansion of clones which should be destined to mature into terminally-differentiated effector cells get locked into endless proliferation with few cells reaching maturation. Treatment with retinoic acid, depending on the precise genomic abnormality, often releases the responsible promyelocytes from this blockade but clinically can yield adverse sequellae in terms of potentially lethal side effects, referred to as retinoic acid syndrome.

**Results:**

Briefly, the list of genes for temporal patterns of expression was pasted into the ABCC GRID Promoter TFSite Comparison Page website tool and the outputs for each pattern were examined for possible coordinated regulation by shared regelems (regulatory elements). We found it informative to use this novel web tool for identifying, on a genomic scale, genes regulated by drug treatment.

**Conclusion:**

Improvement is needed in understanding the nature of the mutations responsible for controlling the maturation process and how these genes regulate downstream effects if there is to be better targeting of chemical interventions. Expanded implementation of the techniques and results reported here may better direct future efforts to improve treatment for diseases not restricted to APL.

## Background

Microarray technology has shown great promise for unraveling the many genomic responses of cells to both developmental signals and chemical stressors[1] despite earlier justified concerns for their reliability[2]. In diseases such as cancer, the confluence of dysregulated developmental programs and the need for treatments to either repair or eliminate dysfunctional cells suggests a valuable role for microarray measurement of gene expression. Knowing which genes are active in a cancer can allow targeting of biochemical pathways to either trigger cell death or to promote restorative gene responses[3] such as terminal differentiation.

Manifold pathway analysis tools are being provided by leaders in microarray data analysis. However, the mere provision of a pathway map selected from genes differentially expressed in an experiment may not provide substantive understanding of their regulation in such a way that provides much guidance in terms of transcriptional control of the genes. What is lacking is the means to identify potentially-approachable targets for subsequent experimentation. Transcriptomics is gene expression profiling for RNAs expressed by a genome at a given time[4]. Recent studies have shown complex involvement of transcriptional regulators in cells' genomic response to stress, growth factors, and even metabolic adaptation[5,6]. Considerable work has been devoted to working out straight-line pathways of signal transduction. As the work has progressed, it is abundantly clear that many genes have multiple regulatory elements (regelems) under the control of sequence-specific activators and repressors of the core RNA polymerase II (Pol II) complex[7], including CBP and the related p300 protein as essential co-activators of the retinoic acid receptor[8]. Indeed, a recent update to a database of known regelems in mammals added about 500 to the previous list of 4900[9,10]. We undertook to examine coordinated gene expression at the transcriptional level using a cell culture-based model of leukemia.

Mature white blood cells are differentiated cells with limited proliferative potential and short life spans compared with other cells. In the case of leukemia, it is a precursor of one of the types of white blood cells which is responsible for the cancer. Myeloblasts, for example, are an early population of committed progenitors with limited mechanisms that might be described as invariant, in which a stem cell gives rise, through an asymmetric cell division, to one stem daughter and one daughter that undergoes differentiation with limitations to the number of rounds of division within the transit amplifying population[11]. In APL, all-*trans *retinoic acid is used both experimentally and clinically to push cells beyond the differentiation blockade to re-enter the maturation process, becoming neutrophils within days.

Previous work[12] compared expression profiles between two promonocytic leukemia U-937 cell lines and in the current study extended this research on neutrophilic differentiation by performing time-course microarray analysis on all-*trans *retinoic acid (ATRA)-treated subclones. The subclones had previously been characterized as either supportive (referred to as "Plus"; subclone 10) or non-supportive ("Minus"; subclone 17) of HIV infection[12], the Minus cells having been found to be at a more differentiated stage with lower telomerase activity. Both subclones, as with U-937 cells in general, respond to treatment with ATRA by resuming differentiation, with concomitant nuclear condensation, development of heterochromatin, production of neutrophilic granules, and expression of cell surface markers CD11b and, chronologically, CD11c. We sought to examine more in-depth the microarray analysis of the transcriptional changes taking place during this process of differentiation.

The output from microarray studies generally is in the form of a spreadsheet listing gene identifiers, their relative levels of expression and additional tracking data. For chronosequential time-course studies, groups of genes exhibiting contemporary modulation of expression by >2-fold at any given time point are placed into groups depending on their pattern, such as up-up-up (indicated as U-U-U), down-down-down (D-D-D), etc, over time. Transcription factors in such groups of genes may be responsible for regulation of the genes in that group[13]. When there is likelihood that treatments may modulate expression of co-regulated genes, it can be desirable to examine the upstream regulatory sequences for the occurrence of matching regulatory elements. To perform regulatory element analysis, grouping of the genes based on the patterns of expression is reasonable[14]. By employing transcriptomic regelem analysis[15], we sought to map potentially important regulatory sites common in genes coordinately modulated. The resultant information steers the experimenter toward the goal of gaining control over the differentiation process in leukemia and other diseases of developmental dysregulation.

## Methods

### Chemicals, reagents, etc

Except as noted, all chemicals and other reagents were purchased from Sigma Chemical Company (St. Louis, MO). All-*trans*-retinoic acid (ATRA) was provided by the drug repository of NCI's Developmental Therapeutics Program (Rockville, MD). The compound was prepared in aliquots, and kept frozen at -70°C until required.

### Cell culture (time-course study)

By convention[16], expanding cultures of U-937 were grown in 75 cm^2 ^tissue culture flasks (Costar #430641 flasks, Corning-Costar, Corning, NY) for 3 days, harvested and plated in 15 ml of complete RPMI-1640 (cRPMI; 10% FBS [Hyclone, Logan, UT], 2 mM *L*-glutamine, and 10 U/ml\10 μg/ml Penicillin\Streptomycin [others GibcoBRL, Grand Island, NY] at a seeding density of 2E5/ml. After 24 hrs, these freshly-passaged cells were counted and replated in 24-well plates (Costar #3527, Corning-Costar) at 1 ml/well at the same density. After another 24 hrs, the wells were dosed with 10 μM ATRA at a final volume of 50 μl in cRPMI added to the 1 ml culture and allowed to incubate for up to 2 days.

### Microarray analysis

The human monoblastoid cell line U-937 subclones Plus and Minus were stimulated to differentiate with 10 μM ATRA and were studied in duplicate samples using the 12 k Affymetrix HG-U95Av2 microarrays (Affymetrix, Santa Clara, CA). The RNA was isolated, purified, and labeled according to the protocol for the chip (*Eukaryotic Sample and Array Processing*, Tech. Man. 701024 Rev. 2., Affymetrix). Each subclone was represented by 4 microarrays, with duplicate subsamples taken at 0, 6, 24 and 48 hours, for comparison within and between the subclones. The human genome U95A array from Affymetrix (Santa Clara, CA) which contains probes interrogating approximately 12,000 full-length genes was employed for the microarrays. Samples from different preparations of the same clone were independently prepared and analyzed following the manufacturer protocols.

### Data analysis

The chronosequential nature of the microarray data [see Additional files 1] permitted clustering of the results by the temporal patterns of gene expression for genes modulated by >2-fold differences [see Additional files 2 and 3]. Genes with similar patterns of expression can naturally be organized together[14]. Groups of genes that fit patterns such as up-up-up, up-up-down, etc. ordered in the Plus subclone did not match precisely with the Minus subclone. Hence, the 8 possible groupings (U-U-U, U-U-D, etc.) had to be treated separately for each subclone. These groups were then submitted for regelem analysis. Briefly, the groups of genes are loaded into DAVID[17] (Database for Annotation, Visualization and Integrated Discovery) using "Upload New List", with "Annotation Tools" selected, and "RefSeq" chosen as the output. The resultant RefSeq identifiers are then copied and pasted into the Advanced Biomedical Computing Center (ABCC) site [18](following the Quick Reference instructions at that site). The output consists of the positions of regulatory elements for each of the genes, in a format conducive to copying and pasting into a spreadsheet where they are examined for shared promoters. This process provides a means to examine groups of genes for expression changes which may be co-regulated by shared regelems.

### Databases

For each input gene or accession number, the ABCC GRID Promoter Comparison Page accesses a pre-computed database of consensus TFSite matches within upstream regions (bases from -1500 to +200) as extracted relative to the coordinates of that gene in the selected genomic sequence. The coordinates of each gene/accession number were derived from the UCSC database files for RefGene entries and the TFSite consensus sequences were taken from the TFSites.dat database file from IFTI[19] (Institute for Transcriptional Informatics). In cases where gene names are supplied rather than accession numbers, the first matching accession number corresponding to that gene name is taken. This procedure is repeated for each of the gene names or accessions in the users' list and a matrix is derived for each transcription factor consensus binding site and each gene. This matrix is then filtered to return only those sites matching at least the selected number of genes in the list. The probabilities of the consensus site matches were approximated from the base composition of all of the promoter regions and the actual sequence and the reciprocal of this number represents the approximate number of bases that would be expected to contain a match to the consensus sequence. A regular expression method was used to identify these sites and thus the degenerate nucleotides present in the consensus sequences are also matched. A more sophisticated method that uses probabilistic methods and profiles is under development, but there are far fewer of these profiles available than the consensus sequences. Also, although this method only identifies pre-defined sequences, a separate utility in the Promoter analysis portion of the GRID web site allows the user to either search all promoter sequences for a user-defined consensus sequence, or to identify short words conserved in a set of promoter sequences (or other user-input sequences).

## Results

**Figure 1:**
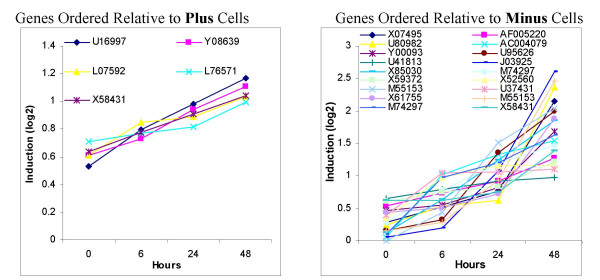
**"Up-Up-Up" Temporal Pattern of Microarray Gene Expression in U-937 Cells**. The figure demonstrates the clustering of genes sharing the pattern of steadily increasing expression level for RNA following treatment with ATRA as measured by microarray. Two subclones of U937 cells are shown, "Minus" and "Plus", over the time course of 0, 6, 24, and 48 hrs. [For all eight clusters for both subclones see Additional File 2.]

A model of leukemia was examined using microarray results organized as clusters of coordinately-regulated genes which changed expression level in response to a drug treatment, i.e., groupings of genes up- or down-regulated in coordinate temporal patterns (Figure 1). Previously, it had been reported[12] that complex interrelationships exist between numerous regulators for two subclones of U-937 monoblastoid cells which did or did not support HIV infection (referred to as Plus cells and Minus cells, respectively). Further studies by the same team went on to study differentiation in U-937 cells by performing microarray analysis on 10 μM ATRA-treated cells with isolation of RNA at 4 time points (0, 6, 24, 48 hrs). The Minus cells exhibited shorter telomeres, less telomerase, and altered gene expression and this was related to the relative maturation state of the cells, with the Minus cells being further differentiated than the Plus cells. Lower c-MYC expression was associated with the reduced telomerase activity in the Minus cells, suggesting involvement of c-MYC in the regulation of telomerase, leading to a predictive model for telomerase therapy[20]. Down-regulation of c-MYC is a hallmark of granulocyte differentiation and is seen in HL-60 cells treated with ATRA[21,22].

It was found that 684 genes have both a significant t-test (*p *< 0.05) and a Positive call in the detection analysis for both replicates with 156 and 238 genes more than two-fold up-regulated or down-regulated, with the regulated genes involved in a variety of cell functions, including proliferation, differentiation and apoptosis, cytoskeletal organization, enzymatic activities and signaling through receptors [see Additional file 1]. The results compared favorably with 14 ATRA-treated U-937 genes reported as up-regulated at 16 hrs in another study[23] also being called as up-regulated in our results at both 6 and 24 hrs. The data, when clustered as patterns of gene expression over time, yielded groups of genes with patterns described as up-up-up (from time point 0 hours to 6 hours to 24 hours to 48 hours and abbreviated UUU), up-up-down (UUD), et cetera for the eight possible combinations. The numbers of genes in each group varied, as did clustering for Minus versus the Plus cells. The identical genes when sorted for the opposite subclone showed irregular, unclassifiable patterns (Columns 1 and 4 in Figure 1).

### "Hourglass" analysis

**Figure 2:**
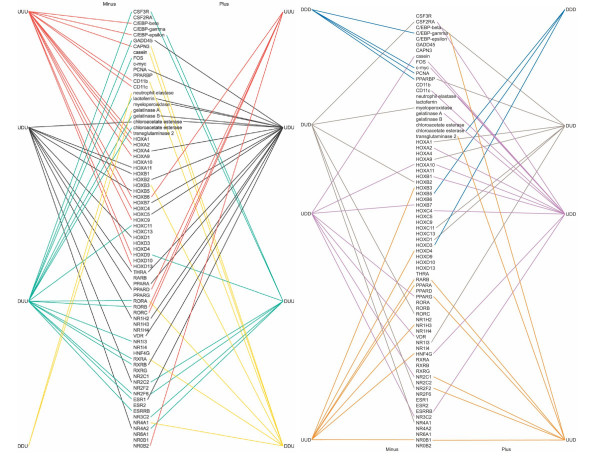
**"Hourglass" Diagram**. Differences in gene expression microarray patterns between Plus and Minus subclones (right and left of both spines, respectively) of the U-937 monoblastoid cell line treated with all-trans retinoic acid over time. Eight expression patterns (four on the left panel and four on the right panel) were defined (up-up-up [UUU], up-down-up [UDU], etc.) for genes of interest related to differentiation: HOX genes, nuclear receptor genes, and genes associated with differentiation in neutrophils. Visualization of the connection differences on either side of the "spine" gene list suggests differential regulation of gene expression in the two subclones for each of the gene clusters.

The patterns of gene expression for the two U-937 subclones, Minus and Plus, were compared graphically (Figure 2). Three types of gene categories were selected based on their described involvement in maturation of eukaryotic cells in general (HOX family and nuclear receptors) or in neutrophils in particular (specific-function genes). Broadly, the genes shown for each expression pattern exhibit a few similarities but there are far more differences (Table 1).

**Table 1:** Similarities and differences in gene expression between U-937 Minus and Plus subclones. Eight chronosequential patterns of gene expression in Minus and Plus subclones shared relatively few genes within clusters of coordinately-expressed genes, even when accounting for the varied numbers of genes expressed for each pattern-subclone combination. Differences quantitatively were greater than similarities.

	Genes in Subclone		Similarities	Differences	
	Minus	Plus		Minus	Plus

UUU	14	5	1	13	4
UDU	10	16	3	7	13
DUU	15	7	2	13	5
DDU	2	7	0	2	7
DDD	4	2	0	4	2
DUD	6	7	0	6	7
UDD	7	12	0	7	12
UUD	8	9	3	5	6

Specifically, note for Figure 2 that there are 38 HOX genes, 72 nuclear receptor genes and we had an original list of 32 neutrophil-related genes of interest. Of these numbers, the Affymetrix GeneChip HumanGenome-U95Av2 only probed 19, 16, and 23, respectively. Of these 58 genes, 7 (CSF3R, HOXC13, HOXD10, NR1H4, RXRG, ESR2, and NR6A1) did not fall into one of the 8 expression pattern cluster categories (UUU, UDU, etc.) by not being induced or repressed by >2-fold. Thus, there are 51 genes plotted in Figure 2.

The eight empirical categories revealed some interesting distinctions between the putatively more mature Minus versus Plus cells for the 50 genes of interest. The Plus cells exhibited no UUU gene expression for any of the neutrophil-specific genes in the top portion of the left hourglass figure. On the other hand, the Minus cells showed induction of genes for the transcription factors C/EBP-beta and C/EBP-epsilon, the intracellular protease calpain 3, the integrins CD11b and CD11c, and transglutaminase 2, involved in apoptosis and known to be induced by retinoic acid. For the UUU category relative to HOX genes, only HOXB6 was induced in the Plus cells, whereas 8 HOX genes were activated in the Minus cells, including HOXB6. On the other hand, none of the nuclear receptors were induced in the Minus cell genes which have an UUU pattern. Orphan retinoic acid receptors RORB and RORC, transcriptional enhancers which bind hormone response elements, along with NR0B2, a transcriptional repressor, and PPAR-delta, another repressor, were in the UUU cluster for Plus cells. These differences support the researchers' previous conclusion that the Minus cells represent a latter stage of differentiation[12].

The UDU category was bimodal in this time series and, therefore, more difficult to interpret but the Minus cells showed only a single neutrophil-specific gene in this category, chloroacetate esterase, with 4 HOX genes and 5 nuclear receptors. The Plus cells had GADD45, the prototypical DNA-damage response gene, along with PCNA (proliferating cell nuclear antigen) and several genes specifically related to the function of normal neutrophils, lactoferrin, gelatinase B, and chloroacetate esterase. The Plus cells further had 4 HOX genes following the UDU pattern, along with 4 nuclear receptors.

The DUU category was characterized, for the Minus cells, with a pattern more similar to the Plus cells' UDU in that GADD45, gelatinase B, and a chloroacetate esterase followed the DUU pattern, along with FOS, part of the AP-1 transcription factor, and Colony Stimulating Factor 3 Receptor (CSF3R), mutations in which are associated with severe congenital neutropenia. Only a single HOX gene was represented in the Minus column but 8 different nuclear receptors fell into this category. For the Plus cells, C/EBP-epsilon and CSF3R were the only genes from the functional part of the gene list, and just one HOX member, HOX9D, the precise function for which is currently unknown but is at least partially involved in distal development as 5' mutations or complete deletion leads to limb and genital abnormalities. Four nuclear receptors were in the category for the Plus cells.

The DDU category, genes whose expression presumably is repressed and then released, for the Minus cells was represented by only two neutrophil enzymes, neutrophil elastase, and gelatinase A. For the Plus cells, however, calpain 3, casein, and CD11b were in this group, along with HOXB3 and 3 nuclear receptor genes.

In the right hand hourglass figure, again there was the case of a dramatic difference between the gene expression patterns of the two U-937 subclones. For the DDD category for the Minus subclone, there were 4 genes, all in our list of genes of special interest to the study of neutrophil differentiation in response to drug treatments for APL: C/EBP-gamma, which may cooperate with FOS to bind PRE-I enhancer elements; c-Myc, known to be down-regulated by retinoic acid; PCNA, and PPARBP (PPAR binding protein), which, along with TFIID, can activate the SP1 transcription factor, interact with thyroid hormone receptor, or function with p53 in apoptosis. The Plus cells, on the other hand, only included in this category a chloroacetate esterase and two HOX genes.

The bimodal DUD category also showed absolute differences between the subclones in that the Minus cells include two neutrophil function genes, CSF2RA and myeloperoxidase along with three nuclear receptors while the Plus cells had the more transcriptionally-involved C/EBP-beta and PPARBP along with 4 HOX genes and one nuclear receptor gene, NR1I3, associated with both transcriptional regulation and androgen receptor function.

The UDD pattern for the Minus cells indicates that casein was induced and then repressed with two HOX genes associated with late developmental expression and 4 nuclear receptors while the Plus cells showed a complex picture of initial up-regulation followed by down-regulation of genes for transcriptional regulators such as FOS and c-MYC and functional genes including CSF2RA, CD11c, and gelatinase A. Four HOX genes and 2 nuclear receptors also fell into this category.

The last category to be reported, UUD, included no neutrophil function genes for the Minus subclone, unlike the UUU category. There were two HOX genes included and 6 nuclear receptors, three of which were shared with the Plus subclone. C/EBP-gamma was included in the UUD category for Plus cells, along with transglutaminase 2. This is curious as C/EBP-gamma was in the Minus DDD category while transglutaminase 2 was in the Minus UUU. Such results suggest that the Plus cells are not simply developmentally delayed compared to the Minus subclone but may have a differentiation program altered in substance as well as in chronology.

### Regulatory element analysis

The differences between the two subclones of U-937 seemed a fair opportunity to compare in closely parallel systems the potential for identifying *in silico *the regelems likely involved in the differential regulation during the process of differentiation. Hence, we applied the strategy of searching for the regelems common to the genes within each of the 8 expression pattern categories. Briefly, the list of genes for each pattern was pasted into the website tool[18] and the outputs for each pattern were examined for possible coordinated regulation by shared regelems. An example of a typical output list of shared regelems is shown in Table 2 for illustration purposes.

**Table 2:** Comparison of shared transcriptional regulatory elements for 8 gene expression patterns for U-937 Minus and Plus subclones. The top 5% of the resultant output table of regulatory elements are shown below. The entire table can be viewed enlarged in the Supplemental Material. The second row shows the number of elements in the cluster identified by pattern in the top row with the names of potential transcription factor binding sites in the first column. The entire output table represents a resource for interrogating possible involvement of regulatory pathways controlling expression of genes within and between each cluster.

Cluster	UUU-	UUD-	UDU-	UDD-	DUU-	DUD-	DDU-	DDD-	UUU+	UUD+	UDU+	UDD+	DUU+	DUD+	DUD+	DDD+
Factors	14	26	23	42	9	20	133	58	85	32	16	6	44	20	26	74
A-MuLV_US1		x					x	x	x	x						x
A-MuLV_US1!		x														x
AP-2_CS3!									x							
AP-2_CS4							x									
AP-2_CS4!				x				x					x			
AP-2_CS5							x	x		x						
AP-2_CS5!													x	x		
AP-2_CS6		x		x			x			x	x		x			
AP-2_CS6!		x	x	x		x	x	x	x			x	x		x	x

Each inputted gene in the cluster was found within the database. The resultant output table shows all regulatory elements (regelems) in the search range near the transcriptional start site that are shared by all of the genes in the cluster, along with the positions [see Additional file 4 for the details for cluster UUD]. Some genes in this example have multiple sites for a given regelem. The co-occurrence of regelems suggests that one or more may have a role in the regulation of transcription triggered by the drug exposure. Particularly in the case of ATRA or other compounds which may have direct interaction with DNA, the possible involvement of regelems with such drugs may indicate DNA or nucleotide binding activity or, alternatively, interaction with the cognate transcription factor for a given site.

Additional file 5 shows the regelems shared by the genes for each of the 8 pattern clusters for the Minus and Plus subclones, simplified by the removal of the site locations. This allows comparison between subclones or between patterns to visualize regelems possibly giving rise to that pattern. Occham's Razor would suggest that constant induction (U-U-U) or repression (D-D-D) might be controlled simply by constant binding levels (on-off) of transcription factors to their cognate binding sites, with the other patterns modulated by the dynamic binding (rheostat) of one or more TFs. Certainly, more complicated scenarios can be conceived. Nevertheless, these results provide the researcher with the opportunity and direction to take the information back to the lab bench to determine whether interdiction, either through DNA binding or TF interference, might be key in gaining control over the regulation of specific patterns of gene expression and differentiation.

## Discussion

Proliferation and differentiation are opposing ends of a phenotypic spectrum determined by gene expression, largely regulated by transcription factors. To better understand how these patterns of gene expression might be transcriptionally regulated, we developed a means to search a large database of regulatory elements using all of the similarly induced or repressed genes for a given group. The goal was to look for common regulatory elements which might have a role in the gene expression altered by the treatment. Experimentally-derived gene expression clusterings seem especially suitable for such analysis as the genes are more likely to be responding to specific signaling pathways as apposed to broader situations such as homeostatic expression seen in normal development or in developed cancers. The transcription factor search capability examines the 1500 bases immediately upstream and 200 bases downstream of the reported transcriptional start site for the RNA in question. A batch analysis sorts output, making it easy to visualize any regulatory sites shared across the group of genes.

**Figure 3:**
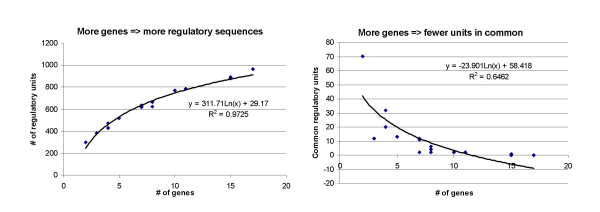
**Plots of the relationship between the number of genes in the clusters for this study with the concordance of regelems**. Regression analyses demonstrate the correlation between the number of genes and the total number of regulatory sequences detected and that the greater the number of genes, the fewer sequences are shared. In other words, the more genes clustered for regelem analysis, the more total number of regulatory elements are involved and the fewer regulatory elements are likely shared in common with increasing numbers of genes. The points represent the 14 gene clusters of the differentiation data set described in the Results.

We have tried to see whether commonalities in the existence of regulatory elements upstream of transcriptional start sites with genes clustered by various techniques may be useful to guide drug development, mechanistic studies, and structure/function relationships in microarray gene expression results. While only limited wet chemistry has been applied to validate such an approach to date, patterns of gene expression must have some rational basis and our analyses seem to point toward a useful role for promoter analysis in extending gene expression analysis beyond simple categorization of groups of genes into the realm of defining the new experiments needed to take control of gene expression in the clinic. Several caveats are clearly warranted, however. One is that the number of genes entered into the current system inversely controls the likelihood of regelems being common to all of the genes (Figure 3A). As a corollary, the more genes entered into the analysis, the more different transcription factors will be represented at some level of frequency (Figure 3B). Deviations from the prediction plots might point to unique characteristics of certain clusters, perhaps involving histone deacetylases or other epigenetic involvement or major control by small interfering RNAs.

With redundancy or near-redundancy more of a rule than an exception in biology, the likelihood exists that regulation of gene expression is provided by the ability of closely-related transcription factors to substitute for others. These complexities may make regulatory element analysis more complex but certainly not unapproachable, thanks to sophisticated statistical techniques such as hierarchical clustering and Self-Organizing Maps. It will be especially worthwhile to collect the experiences of those pursuing such analytical techniques to compile further examples of where the approach is applicable and situations in which it might not be useful. Currently, we hold that short-term, specific-stimulus gene expression studies may benefit most, whereas simple compilations of gene lists thought to be regulated in particular pathways may have less coordinate regulation.

Our method is similar to a large number of efforts to benefit from the mechanistic link between regelems and transcription. In addition to alternative transcription factor databases such as TESS[24] and others[10], there are other outstanding regelem analytical tools. One set of tools in particular, CARRIE and ROVER[13,25], seems particularly competitive with our approach and a web-based version is anticipated. Rover is helpful for determining if one or more of a group of transcription factors is likely to regulate a group of genes based on their over-representation in a group of sequences. CARRIE uses two-condition microarray data and applies promoter analysis to infer the stimulated/repressed transcriptional regulatory network. As of this writing, however, CARRIE has only been validated on yeast and ROVER requires UNIX/Linux-based computational environments and expertise, less "user-friendly" than the facility provided by the ABCC.

The fact that all genes within any given group do not share a single or combination of regelems is not necessarily contrary to our concept of regelem analysis. Related transcription factors may substitute, incrementally modulate, or confer cell-type specificity, allowing more than one regelem to help regulate transcription[7]. For example, stem cells find various means to advance development[11]. This especially might be true for leukemic cells which, if pressed, can hurdle developmental blockades to continue with the maturation program. The probabilities accompanying the regelem outputs help guide confidence in the likelihood of shared regelems potentially having a role in the expression for a group of genes and may help bring to full fruition the field of transcriptomics.

## Availability and requirements

The ABCC GRID Promoter TFSite Comparison Page website is available without restriction and functions with all web browsers tested.

## Added note

Following the preparation of this report, important microarray studies of AML have come to the attention of the authors that merit mention (included in References), one by Zheng *et al*[26] and one by Meani *et al*[27]. Also, the raw data have been made available online[28].

## Disclaimer

The content of this publication does not necessarily reflect the views or policies of the Department of Health and Human Services, nor does mention of trade names, commercial products, or organizations imply endorsement by the U.S. Government. This project has been funded in whole or in part with Federal funds of the Intramural Research Program of the National Cancer Institute, National Institutes of Health, including Contract No. NO1-CO-56000.

## Supplementary Material

Additional File 1 Microarray data for genes with greater than 2-fold differences in expression (up or down) compared to sham-treated controls. Click here for file

Additional File 2 Temporal Patterns of Microarray Gene Expression in U-937 Cells. Click here for file

Additional File 3 Temporal Patterns of Microarray Gene Expression in U-937 Cells (Continued). Click here for file

Additional File 4 **Example of regulatory element analysis output for gene cluster Up-Up-Down of Plus subclone of U-937 cells exposed to ATRA with 4 time point samples over 48 hrs**. On left are transcription factor binding sites. The sequences are in the second from right-most column, next to the Bayesian probability for that number of nucleotides. Each intervening column shows the positions relative to the transcriptional start site of each regulatory element under the gene name and RefSeq ID of the mRNA. Click here for file

Additional File 5 **Comparison of shared transcriptional regulatory elements for 8 gene expression patterns for U-937 Minus and Plus subclones**. The top row shows the number of elements in the cluster identified by pattern in the second row. Because the number of elements is inversely proportional to the number of genes in the cluster, the fact that only 2 genes occur in the Minus cluster DDU-results in 133 elements in the output, which is not particularly informative for this cluster. Click here for file

## References

[B1] Schena M, Shalon D, Davis RW, Brown PO (1995). Quantitative monitoring of gene expression patterns with a complementary DNA microarray. Science.

[B2] Kothapalli R, Yoder SJ, Mane S, Loughran TPJ (2002). Microarray results: how accurate are they?. BMC Bioinformatics.

[B3] Gunther EC, Stone DJ, Gerwien RW, Bento P, Heyes MP (2003). Prediction of clinical drug efficacy by classification of drug-induced genomic expression profiles in vitro. Proc Natl Acad Sci U S A.

[B4] Anon. (1999). Proteomics, transcriptomics: what's in a name?. Nature.

[B5] Shi YH, Fang WG (2004). Hypoxia-inducible factor-1 in tumour angiogenesis. World J Gastroenterol.

[B6] Davis CD, Milner J (2004). Frontiers in nutrigenomics, proteomics, metabolomics and cancer prevention. Mutat Res.

[B7] Mannervik M, Nibu Y, Zhang H, Levine M (1999). Transcriptional coregulators in development. Science.

[B8] Eckner R, Ewen ME, Newsome D, Gerdes M, DeCaprio JA, Lawrence JB, Livingston DM (1994). Molecular cloning and functional analysis of the adenovirus E1A-associated 300-kD protein (p300) reveals a protein with properties of a transcriptional adaptor. Genes Dev.

[B9] Ghosh D (2000). Object-oriented transcription factors database (ooTFD). Nucleic Acids Res.

[B10] Galperin MY (2004). The Molecular Biology Database Collection: 2004 update. Nucleic Acids Res.

[B11] Watt FM, Hogan BL (2000). Out of Eden: stem cells and their niches. Science.

[B12] Xiao X, Phogat SK, Sidorov IA, Yang J, Horikawa I, Prieto D, Adelesberger J, Lempicki R, Barrett JC, Dimitrov DS (2002). Identification and characterization of rapidly dividing U937 clones with differential telomerase activity and gene expression profiles: role of c-Myc/Mad1 and Id/Ets proteins. Leukemia.

[B13] Haverty PM, Frith MC, Weng Z (2004). CARRIE web service: automated transcriptional regulatory network inference and interactive analysis. Nucleic Acids Res.

[B14] Eisen MB, Spellman PT, Brown PO, Botstein D (1998). Cluster analysis and display of genome-wide expression patterns. Proc Natl Acad Sci U S A.

[B15] Patton GW, Sidorov IA, Dimitrov DS, Xiao X, Shoemaker RH, Tudor G, Covell D, Stephens R (2005). The ABCC GRID Promoter TFSite Comparison Page to find shared regulatory elements for co-regulated genes. Submitted.

[B16] Sundstrom C, Nilsson K (1976). Establishment and characterization of a human histiocytic lymphoma cell line (U-937). Int J Cancer.

[B17] DAVID (Database for Annotation VID DAVID (Database for Annotation, Visualization and Integrated Discovery). http://david.niaid.nih.gov/david/ease.htm.

[B18] ABCC GRID Promoter TFSite Comparison Page ABCC GRID Promoter Comparison Page. http://grid.abcc.ncifcrf.gov/promoters/comparePromoters.php.

[B19] IFTI (Institute for Transcriptional Informatics). http://www.ifti.org/.

[B20] Sidorov IA, Hirsch KS, Harley CB, Dimitrov DS (2003). Cancer treatment by telomerase inhibitors: predictions by a kinetic model. Math Biosci.

[B21] Cowen DS, Berger M, Nuttle L, Dubyak GR (1991). Chronic treatment with P2-purinergic receptor agonists induces phenotypic modulation of the HL-60 and U937 human myelogenous leukemia cell lines. J Leukoc Biol.

[B22] Xu D, Popov N, Hou M, Wang Q, Bjorkholm M, Gruber A, Menkel AR, Henriksson M (2001). Switch from Myc/Max to Mad1/Max binding and decrease in histone acetylation at the telomerase reverse transcriptase promoter during differentiation of HL60 cells. Proc Natl Acad Sci U S A.

[B23] Park DJ, Vuong PT, de Vos S, Douer D, Koeffler HP (2003). Comparative analysis of genes regulated by PML/RAR alpha and PLZF/RAR alpha in response to retinoic acid using oligonucleotide arrays. Blood.

[B24] Baxevanis AD (2003). Current protocols in bioinformatics.

[B25] ROVER CARRIE CARRIE and ROVER. http://sullivan.bu.edu/~phaverty/#tools.

[B26] Zheng PZ, Wang KK, Zhang QY, Huang QH, Du YZ, Zhang QH, Xiao DK, Shen SH, Imbeaud S, Eveno E, Zhao CJ, Chen YL, Fan HY, Waxman S, Auffray C, Jin G, Chen SJ, Chen Z, Zhang J (2005). Systems analysis of transcriptome and proteome in retinoic acid/arsenic trioxide-induced cell differentiation/apoptosis of promyelocytic leukemia. Proc Natl Acad Sci U S A.

[B27] Meani N, Minardi S, Licciulli S, Gelmetti V, Coco FL, Nervi C, Pelicci PG, Muller H, Alcalay M (2005). Molecular signature of retinoic acid treatment in acute promyelocytic leukemia. Oncogene.

[B28] Patton2005U937.XLS Patton2005U937.XLS. http://home.ncifcrf.gov/research/bja/Patton2005U937.XLS.

