# Supplementary material for: Transcriptomic response to differentiation induction
Source: BMC Bioinformatics. 2006 Feb 17;7:81. doi: 10.1186/1471-2105-7-81 (PMC1395336; doi:10.1186/1471-2105-7-81)
Supplement: Additional File 3 — Temporal Patterns of Microarray Gene Expression in U-937 Cells (Continued). [file 1471-2105-7-81-S3.ppt]

## Slide 1
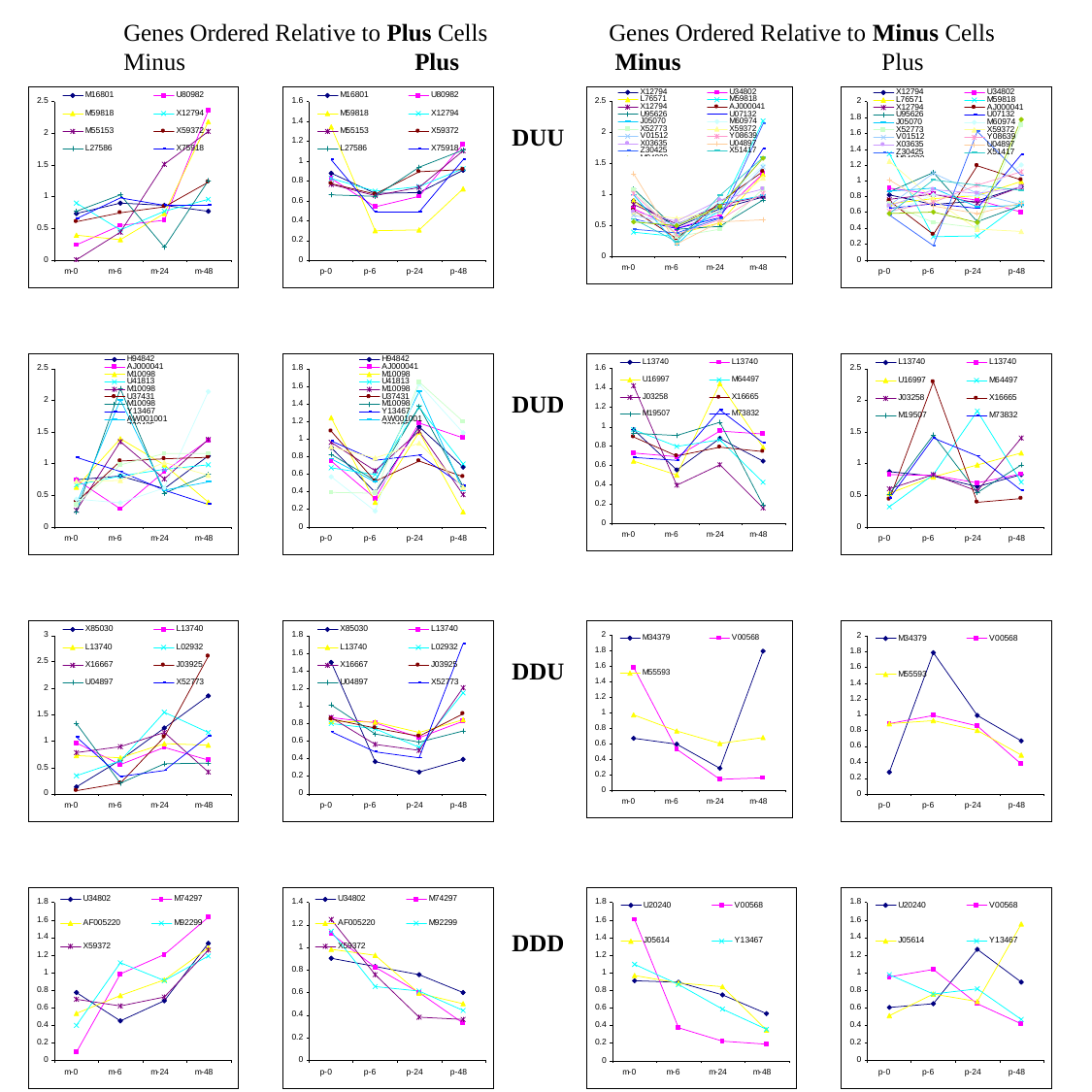

Genes Ordered Relative to Plus Cells Genes Ordered Relative to Minus Cells
Minus		Plus	 Minus		 Plus
DUU
DUD
DDU
DDD
