# Supplementary figures and images for: Transcriptomic response to differentiation induction
Source: BMC Bioinformatics. 2006 Feb 17;7:81. doi: 10.1186/1471-2105-7-81 (PMC1395336; doi:10.1186/1471-2105-7-81)

## Slide 1
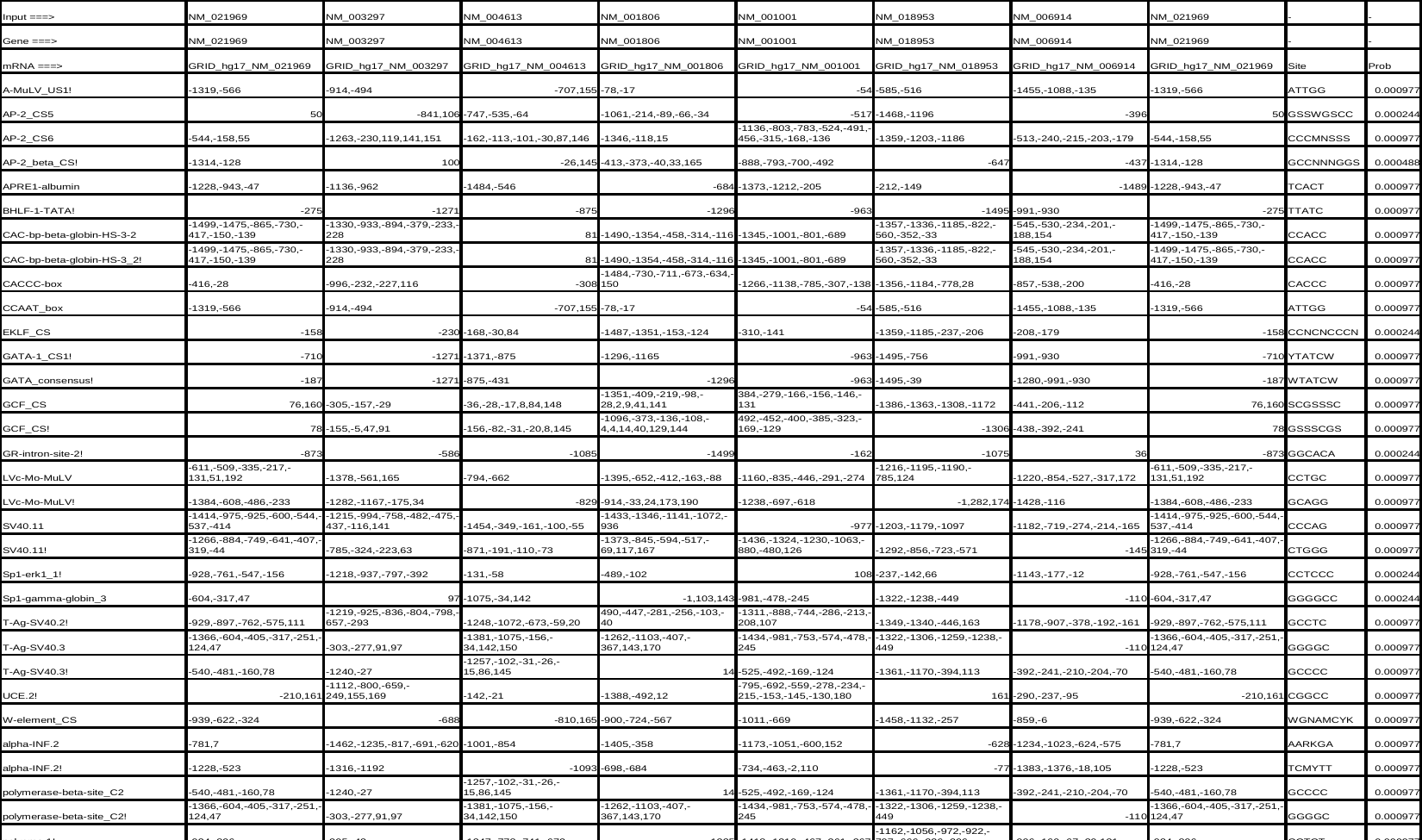

Supplement: Additional File 4 — Example of regulatory element analysis output for gene cluster Up-Up-Down of Plus subclone of U-937 cells exposed to ATRA with 4 time point samples over 48 hrs. On left are transcription factor binding sites. The sequences are in the second from right-most column, next to the Bayesian probability for that number of nucleotides. Each intervening column shows the positions relative to the transcriptional start site of each regulatory element under the gene name and RefSeq ID of the mRNA. [file 1471-2105-7-81-S4.ppt]

## Slide 1
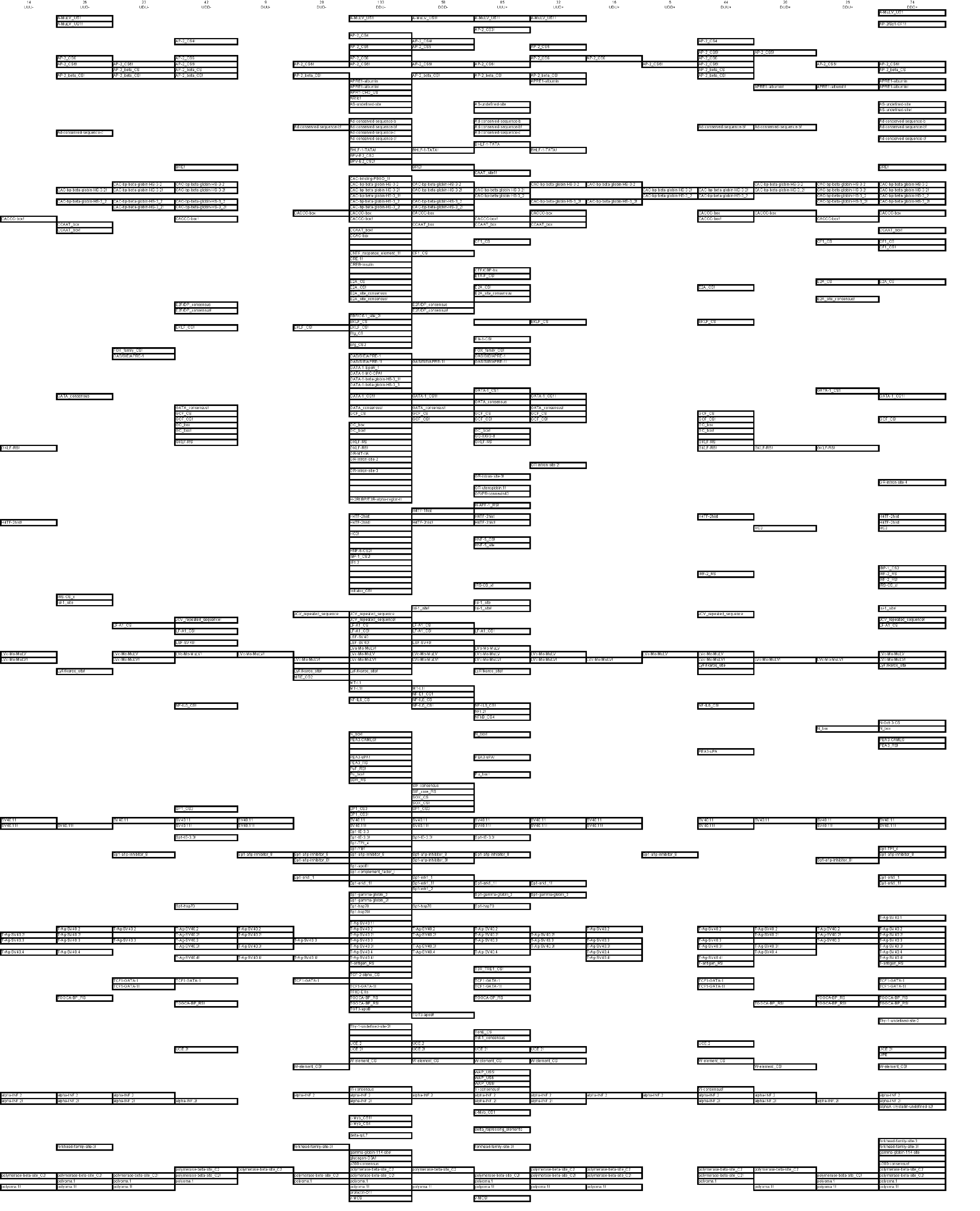

Supplement: Additional File 5 — Comparison of shared transcriptional regulatory elements for 8 gene expression patterns for U-937 Minus and Plus subclones. The top row shows the number of elements in the cluster identified by pattern in the second row. Because the number of elements is inversely proportional to the number of genes in the cluster, the fact that only 2 genes occur in the Minus cluster DDU-results in 133 elements in the output, which is not particularly informative for this cluster. [file 1471-2105-7-81-S5.ppt]
